# Collet‐Sicard syndrome due to concurrent extramedullary intracranial plasmacytoma and jugular venous sinus thrombosis in multiple myeloma

**DOI:** 10.1002/ccr3.4457

**Published:** 2021-07-28

**Authors:** Dilraj Singh Sokhi, Caroline Wangui Mithi, Farah Alnoor Ebrahim, Adil Salyani, Sheila Waa, Malkit Singh Riyat

**Affiliations:** ^1^ Department of Medicine, Faculty of Health Sciences The Aga Khan University Hospital Aga Khan University Medical College of East Africa Nairobi Kenya; ^2^ Department of Radiology, Faculty of Health Sciences The Aga Khan University Hospital Aga Khan University Medical College of East Africa Nairobi Kenya; ^3^ Department of Oncology and Haematology, Faculty of Health Sciences The Aga Khan University Hospital Aga Khan University Medical College of East Africa Nairobi Kenya

**Keywords:** cranial neuropathy, multiple myeloma, plasmacytoma

## Abstract

In a patient with Collet‐Sicard syndrome and multiple myeloma, both extramedullary plasmacytomas and internal jugular vein‐sigmoid sinus thrombosis should be considered as they can coexist.

## Graphical abstract

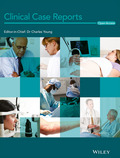

## 1 INTRODUCTION

Multiple myeloma (MM) can cause cerebral venous sinus thrombosis (CVST) or cranial neuropathies. Combined unilateral palsies of cranial nerves IX‐XII are called Collet‐Sicard syndrome (CSS), which can be caused by MM extramedullary plasmacytomas (MM‐EP) or CVST. Our case report illustrates a unique combination of MM‐EP and CVST causing CSS.

Multiple myeloma (MM) is a malignant clonal plasma cell dyscrasia typically presenting with anemia, hypercalcemia, bony skeletal symptoms, and/or renal failure and comprises 10% of hematological malignancies.^1^ Neurological involvement in MM can occur due to the following^2^: (i) direct infiltration (eg, inflammatory neuropathy); (ii) remote effects of the disease [eg, hypercoagulability causing cerebral venous sinus thrombosis (CVST)]^3^; or (iii) complications from treatment (eg, seizures from melphalan). Central nervous system (CNS) involvement is rare (approximately 1% of MM cases)^4^ and usually due to extramedullary plasmacytomas (EP) from longer‐standing advanced stage MM (MM‐EP). EP comprise 1% of head and neck tumors^5^ and have a predilection for the skull base^6^; intracranial MM‐EP can therefore manifest as cranial neuropathies depending on the site of the lesion.^7^ MM‐EP carry a poor prognosis, with median overall survival of approximately 2 months.^8^

Collet‐Sicard syndrome (CSS) is a rare presentation of combined unilateral palsies of cranial nerves IX, X, XI and XII from skull base lesions that affect both the jugular foramen *pars nervosa* and the hypoglossal canal. In a recent review of 51 CSS cases reported in the literature, 40% are caused by malignancies, including EP, and 33% by vascular disease including CVST.^9^ However, the majority of the reported EP cases causing jugular foramen syndromes such as CSS are solitary plasmacytomas rather than MM‐EP.^10^ There are no reported CSS cases caused simultaneously by both MM‐EP and CVST.

## 2 CASE EXAMINATION

### 2.1 Case history

A 57‐year‐old female initially presented at an external facility with fatigue and anemia. She was found to have a monoclonal band on serum protein electrophoresis and 62% plasmocytosis on bone marrow biopsy which confirmed the diagnosis of MM. She was treated with dexamethasone 40mg weekly and a 21‐day cycle of lenalidomide 25 mg once a day for 6 months. Repeat bone marrow examination revealed no excess plasma cells, and the lenalidomide dose was reduced to 10mg. However, she elected to transfer to our facility a year later after complaining of four months of hoarseness of voice, difficulty in swallowing, and night sweats despite her ongoing treatment.

Initial blood tests revealed normal renal function and calcium level, and the following abnormal results: a normocytic normochromic anemia with a hemoglobin of 7.7 g/dl [normal range (NR) 11.5–16.5 g/dl], monoclonal band on serum protein electrophoresis (0.15 g/dl; NR 0), and beta‐2 microglobulin raised at 7152 ng/ml (NR 670–2143). Serum free light chain (FLC) assay revealed elevated kappa (κ) light chains (4840 mg/L; NR 6.7–22.4) with normal lambda (λ) light chains (12.9 mg/L; NR 8.3–27.0) giving an elevated κ/λ ratio of 375.2 (NR 0.26–1.65). There were no bone lesions on cross‐sectional magnetic resonance imaging (MRI), and a computed tomography (CT) scan of the head was reported as normal.

### 2.2 Differential diagnosis

The investigations were in keeping with suboptimal MM disease control. Her therapy was optimized to the VRD regimen: weekly subcutaneous bortezomib (V) 2.5mg (calculated as 1.3 mg/m^2^ body surface area) once a week for three weeks followed by one‐week break, lenalidomide (R) 25 mg once a day for three weeks followed by one‐week break, and dexamethasone (D) 40 mg once a week. However, her dysphonia, dysarthria, and dysphagia progressed, and she developed generalized body fatigue, weakness and episodes of tetany, which led to an urgent admission a month later.

### 2.3 Investigations

Repeat blood tests revealed worsening anemia with a hemoglobin of 5.0 g/dl, thrombocytopenia (platelet count of 128 × 10^9^/L; NR 150–400), hypokalemia (2.50mmol/L; NR 3.3–5.4), hyponatremia (131 mmol/L; NR 136–145), hypomagnesaemia (0.44 mmol/L; NR 0.66–1.07), hypocalcaemia (1.9mmol/L corrected; NR 2.15–2.55), and hypo‐albuminemia (30.6 g/L; NR 35–55), but normal renal function and insignificantly elevated liver function tests. These results led to the diagnosis of Fanconi syndrome (FS) being caused by light chain proximal tubulopathy (LCPT). We corrected the electrolyte imbalances with the help of the nephrology team, but her neurological symptoms persisted so she was reviewed by the neurology team.

Cranial nerve examination revealed absent gag reflex, left palatal weakness with contralateral uvula deviation, weakness of the left sternocleidomastoid and trapezius muscles, and a weak, wasted and fasciculating tongue that was deviated to the left. Gustatory function in the posterior third of the tongue was difficult to assess, and the rest of the neurological examination was normal. The signs found were all in keeping with combined multiple ipsilateral lower cranial neuropathies consistent with CSS.

**FIGURE 1:**
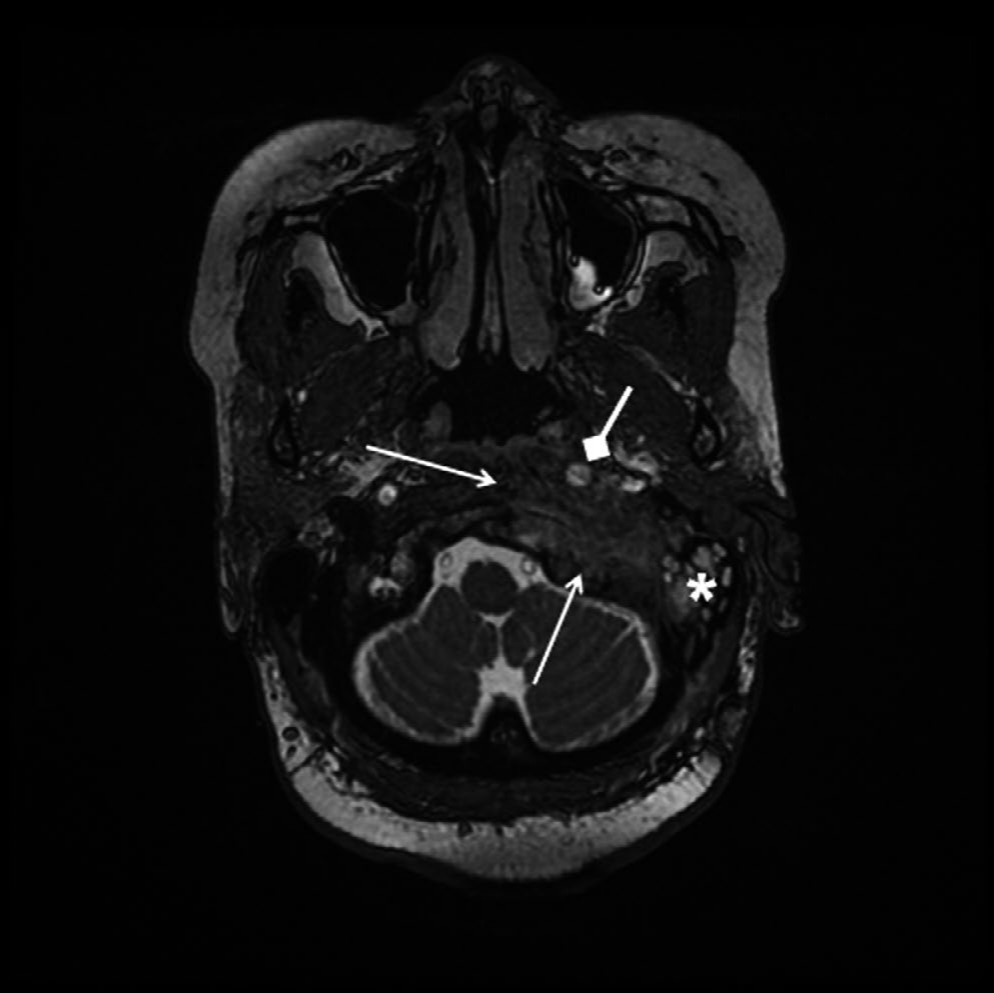
T2‐weighted thin‐slice fast imaging employing steady‐state acquisition (FIESTA) magnetic resonance imaging (MRI) sequence shows left clival and petrous bone T2 hypointense mass (open arrows) displacing the left internal carotid artery anteromedially (diamond arrow), with left mastoid effusion (asterisk)

**FIGURE 2:**
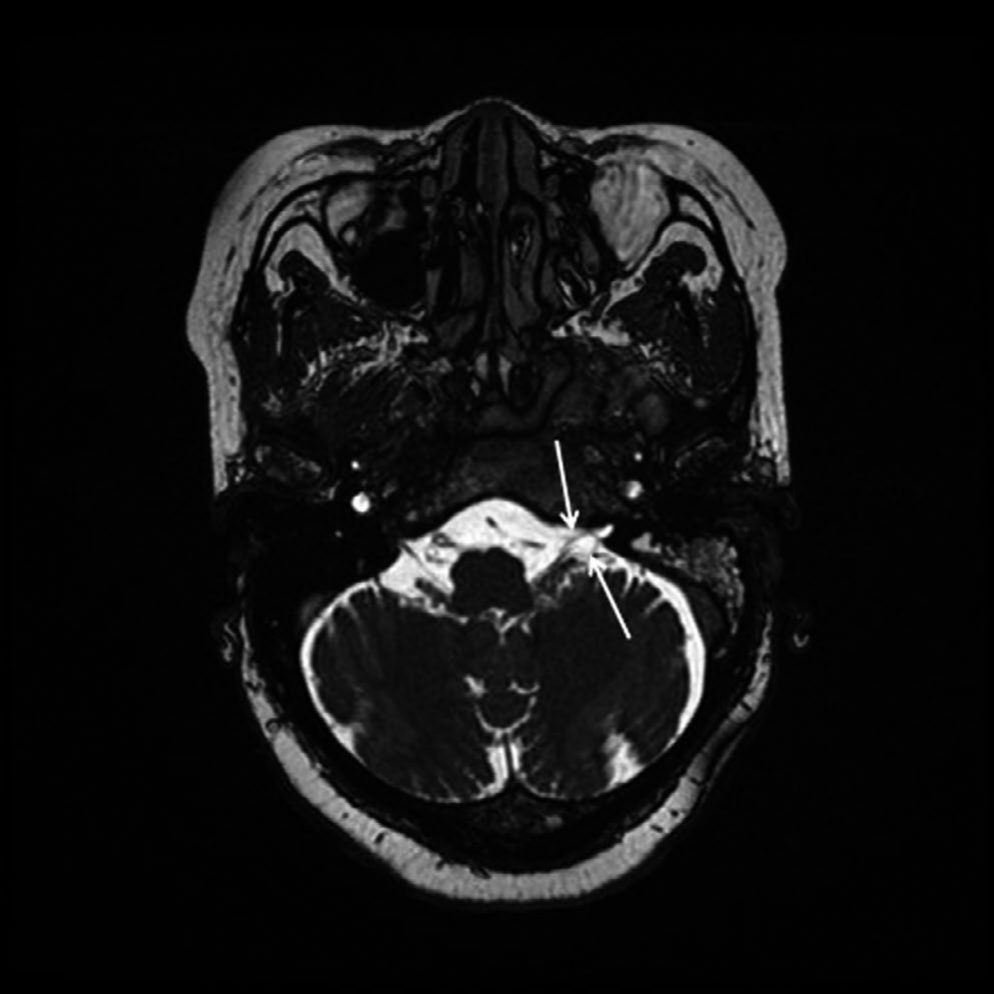
T2‐weighted thin‐slice FIESTA MRI sequence shows thickened left glossopharyngeal and vagus nerves (open arrows) entering a narrowed jugular foramen pars nervosa

**FIGURE 3:**
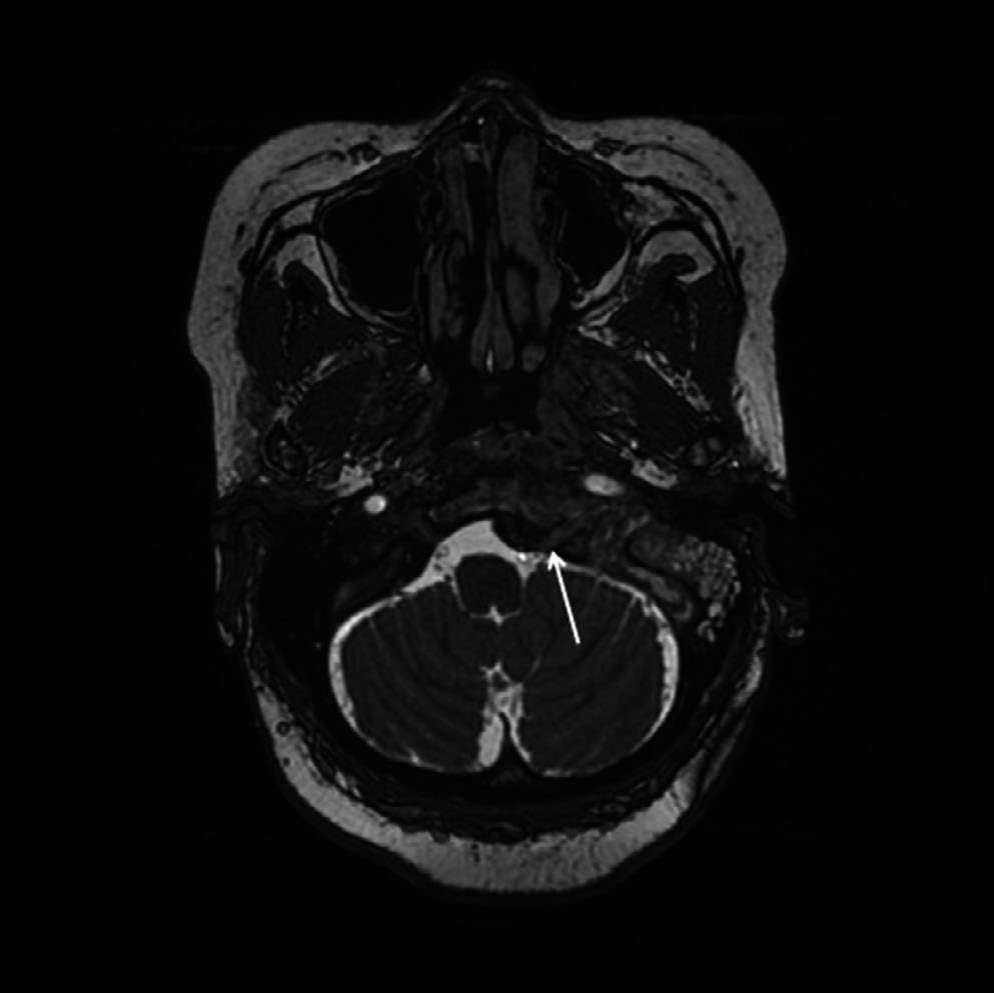
T2‐weighted thin‐slice FIESTA MRI sequence shows a thickened left hypoglossal nerve (open arrow) within hypoglossal canal narrowed by inferior aspect of mass

**FIGURE 4:**
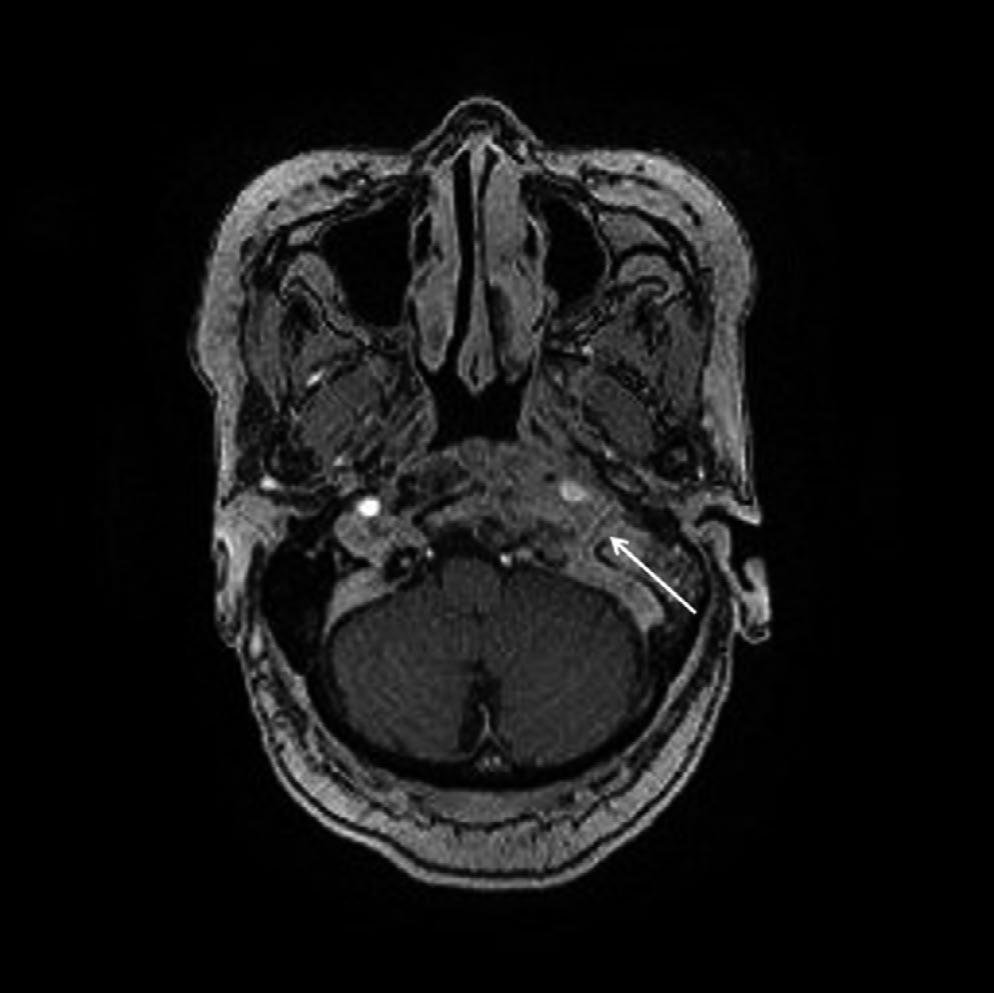
T1‐weighted MRI with contrast showing left clival, prevertebral, and petrous mass obscuring left jugular bulb (open arrow)

**FIGURE 5:**
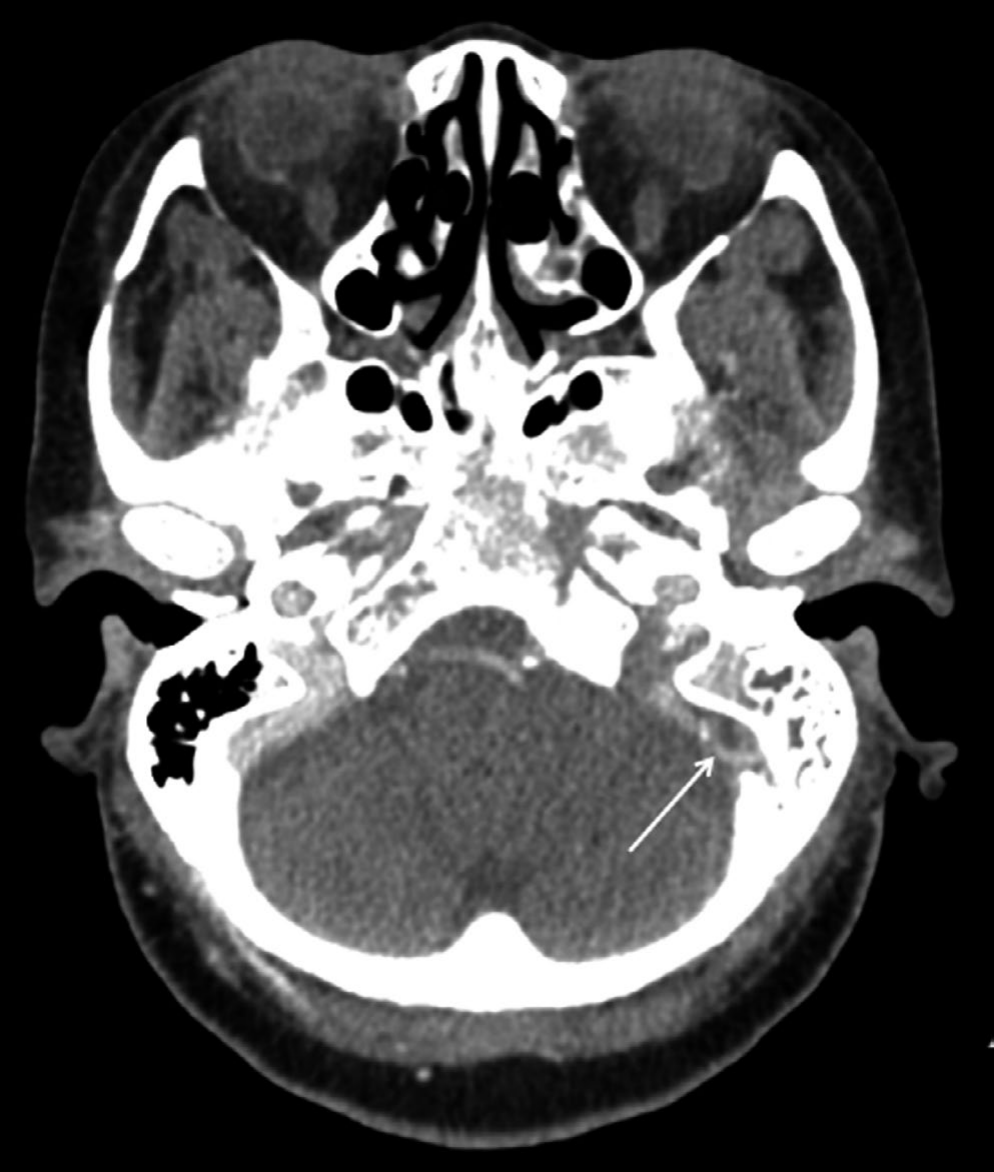
Computed tomography (CT) venography of the intracranial vasculature showing no flow in left sigmoid‐jugular complex (open arrow) in keeping with occlusive cerebral venous sinus thrombosis

MRI brain scan with contrast revealed reduced T1 signal in the marrow of the left clivus, petrous and mastoid temporal bones, with gadolinium enhancement extending to the jugular foramina and hypoglossal canals bilaterally, worse on the left. The findings were in keeping with intracranial MM‐EP. Dedicated imaging of the vasculature with CT venography further confirmed CVST in the left sigmoid‐jugular sinus complex extending to the proximal internal jugular vein (Figures 1, 2, 3, 4, 5).

### 2.4 Treatment

The VRD therapy was intensified for the MM‐EP and continued with good effect such that at 6 weeks κ was 311.0 ml/L, λ was 8.57 mg/L, and κ/λ ratio was much improved at 36.3. The patient was also commenced on rivaroxaban (15 mg twice daily for three weeks followed by 20 mg once a day) for the CVST, as she did not want injections of low‐molecular‐weight heparin (LMWH), and there was no reliable warfarin service near her. She was followed up by the hematology service regularly in our hospital's chemotherapy unit to ensure adherence to the VRD treatment.

### 2.5 Outcome and follow‐up

At 3 months, she had complete resolution of her cranial nerve symptoms with no further complications and had found it easy to comply with both rivaroxaban and the VRD regimen. Repeat neuroimaging at this stage showed partial resolution of the CVST; the MM‐EP had marginally increased from 2.7 cubic centimeters (cc) to 3.1 cc (approximately 14% increase), which qualified as stable disease as per the response evaluation criteria in solid tumors (RECIST) criteria.^11^ We therefore decided to continue the VRD regimen and anticoagulation for a total of 6 months, in line with respective international guidelines.^12^

## 3 DISCUSSION

Our patient did not have the appropriate treatment for MM at the outset, which may have led to early extramedullary disease progression. Disease dissemination usually occurs years later even in stable patients treated with autologous stem cell transplant.^3^ By the time she presented to our facility, she required salvage therapy and as per guidelines we instituted the VRD regimen,^13^ a regimen similar to other regimens such as the carfilzomib‐based KRD (carfilzomib, lenalidomide and dexamethasone),^14^ which have been shown to improve prognosis specifically in disseminated MM including MM‐EP.^15^ There is some evidence that in relapsed or refractory MM patients with MM‐EP, a more intensive “lymphoma‐like” polychemotherapy regimen can be used.^16^ Radiotherapy can offer further prolongation of disease‐free survival,^17^ but our patient's MM‐EP was deemed stable as per the RECIST criteria.^11^ Indeed, there are reports of significant reduction in intracranial MM‐EP and improved prognosis with salvage regimens without the need for radiotherapy.^14^ Surgical treatment is not well established for skull base plasmacytomas, although some recommend salvage surgery in cases of recurrent/persistent disease,^10^, ^18^ which was not pertinent to our case.

Our patient's MM‐EP presented with CSS. CSS can be the first presenting feature of MM,^10^, ^19^ or a presenting feature of dissemination of previously controlled MM^3^; mortality can be within months of such presentations.^20^ Jugular foramen syndromes and CSS are not the only cranial neuropathies that herald MM. Depending on the site of infiltration at the skull base,^7^ MM can first present with: (i) isolated palsies of, for example, the oculomotor,^21^ abducens,^22^ or hypoglossal^23^ nerves; or as (ii) multiple cranial neuropathies that are either adjacent (eg, of the trigeminal and abducens nerve)^24^ or separate (eg, optic neuropathy in combination with other cranial neuropathies).^25^ Even if the presenting feature is cranial, it remains important to look for concurrent neurological involvement in the spinal cord^26^ or peripheral nerves,^27^ which we did in our patient on clinical examination.

The association between MM and FS is well known from the 1950s,^28^ and in adults FS usually occurs due to intracytoplasmic deposition of monoclonal (usually κ) light chains in the proximal tubular epithelial cells, and this leads to LCPT.^29^

Patients with multiple myeloma are at an increased risk of venous thromboembolism, and thromboprophylaxis is recommended, with either aspirin or LMWH depending on disease, patient, and treatment‐related factors.^30^ For our patient, the initiation of appropriate chemotherapy occurred only shortly before the second admission caused by FS, and thromboprophylaxis had therefore not been commenced. It is unlikely our patient developed CVST at this second admission as she did not have headache or visual disturbance, which suggests a more subacute onset of the thrombosis. While the MRI brain scan picked up the MM‐EP in our case, the diagnosis of CVST was only clarified after definitive CT venography. Indeed, dedicated skull‐based CT imaging with additional techniques such as multidetector‐row CT have been shown to adequately diagnose the extent of infiltration and even the cause of CSS.^31^, ^32^ CVST can occur with intracranial MM‐EP,^3^ but only <5% of CVST cases present with multiple cranial nerve palsies,^33^ and CSS caused by CVST has only been described in five cases^2^, ^33^, ^34^, ^35^, ^36^ based on our literature review (see Table 1).

**TABLE 1:** CVST presenting as Collet‐Sicard syndrome

Case report	Age (years), gender	Risk factor	Location of CVST	Duration of anticoagulation
Malin et al, 1984^34^	41, female	Possible slow‐growing tumor	Left transverse sinus	Unclear
Moon et al, 1994^33^	34, female	After an acute febrile illness	Left transverse and sigmoid sinus	2 months
Otto et al, 2001^35^	60, male	Mild IgG light chain paraprotein	Left internal jugular vein	3 months
Handley et al, 2010^36^	30, male	After acute upper respiratory tract infection	Right internal jugular vein and sigmoid sinus	3 months
Neo et al, 2017^2^	71, male	Long‐term central venous catheter for chemotherapy	Left internal jugular vein, sigmoid and transverse sinuses	6 months

Abbreviation: CVST, cerebral venous sinus thrombosis.

Warfarin or LMWH is the recommended treatment for thrombotic events in MM,^30^ and warfarin is the internationally accepted standard treatment of CVST.^37^ However, the risk of bleeding needs to be balanced against the risk of recurrent thrombosis on an individual basis, especially in hematological malignancies where thrombocytopenia increases bleeding risk, especially in the first 6 months of anticoagulation.^30^ Our patient did not have thrombocytopenia at the time of commencing anticoagulation, and as a team we agreed the risk of further CVST or other thromboses was higher in the context of her MM disease being so active, and it is recommended that in such cases anticoagulation should be continued until the underlying disease is controlled.^30^ Warfarin was difficult to institute because of poor local setups for adequate monitoring and dosing, and our patient also did not want injections of LMWH. Direct oral anticoagulants (DOACs) such as rivaroxaban and dabigatran are not recommended because of lack of evidence. For CVST, there is some evidence to show safe and efficacious treatment with rivaroxaban.^38^, ^39^ Only several months after this case have we noted the recently published randomized controlled trial evidence that shows dabigatran is noninferior to warfarin in treating CVST.^40^

Our case benefited from being managed at a tertiary referral center, which had the best diagnostic and therapeutic options available in the region, except for stem cell therapy for which the closest center would have been in South Africa. Our report is limited by the paucity of initial evaluation, including laboratory workup, from the external facility where she first presented in 2018.

## 4 CONCLUSION

MM can affect the nervous system and can manifest in many ways including multiple cranial neuropathies such as CSS, which can even be the first presenting signs of the disease. Patients with MM are more prone to prothrombotic events such as CVST, which can be prevented by timely thromboprophylaxis. Recent advances in MM treatment have improved survival, but MM as a systemic disease can complicate to FS or progress to MM‐EP. Urgent salvage regimens such as VRD can be used to treat relapses and/or progression, resulting in improvement without the need to for surgery or radiotherapy as our case illustrates.

## CONFLICT OF INTEREST

The authors declare that there is no conflict of interest regarding the publication of this paper.

## AUTHOR CONTRIBUTIONS

CWM, FAE and AS summarized the case history and laboratory findings, and developed the background literature review. SW provided the neuroimaging and literature on radiology in Collet‐Sicard syndrome. MSR contributed to and reviewed the hemato‐oncology sections of the case report and background literature. DSS conceptualized the case report and finalized the manuscript.

## ETHICAL STATEMENT

Our work has been conducted in accordance with the Declaration of Helsinki (1964). We have obtained written consent from the patient to publish her case and images. In line with our Institutional Ethics and Research Committee (IERC) guidelines, this case report was exempted from full IERC review.

## Data Availability

The clinical history and imaging data used to support the findings of this study are included within the article.
